# A Lightweight Vehicle Detection Method Fusing GSConv and Coordinate Attention Mechanism

**DOI:** 10.3390/s24082394

**Published:** 2024-04-09

**Authors:** Deqi Huang, Yating Tu, Zhenhua Zhang, Zikuang Ye

**Affiliations:** School of Electrical Engineering, Xinjiang University, Urumqi 830017, China; dqhuang@xju.edu.cn (D.H.); 107552204418@stu.xju.edu.cn (Z.Z.); 107552201556@stu.xju.edu.cn (Z.Y.)

**Keywords:** artificial intelligence, object detection, YOLOv7, lightweight

## Abstract

Aiming at the problems of target detection models in traffic scenarios including a large number of parameters, heavy computational burden, and high application cost, this paper introduces an enhanced lightweight real-time detection algorithm, which exhibits higher detection speed and accuracy for vehicle detection. This paper considers the YOLOv7 algorithm as the benchmark model, designs a lightweight backbone network, and uses the MobileNetV3 lightweight network to extract target features. Inspired by the structure of SPPF, the spatial pyramid pooling module is reconfigured by incorporating GSConv, and a lightweight SPPFCSPC-GS module is designed, aiming to minimize the quantity of model parameters and enhance the training speed even further. Furthermore, the CA mechanism is integrated to enhance the feature extraction capability of the model. Finally, the MPDIoU loss function is utilized to optimize the model’s training process. Experiments showcase that the refined YOLOv7 algorithm can achieve 98.2% mAP on the BIT-Vehicle dataset with 52.8% fewer model parameters than the original model and a 35.2% improvement in FPS. The enhanced model adeptly strikes a finer equilibrium between velocity and precision, providing favorable conditions for embedding the model into mobile devices.

## 1. Introduction

With the continuous growth of the public’s travel demand, the rate of automobile ownership has steadily increased in recent years, leading to increases in traffic accidents and congestion [1]. If accidents and congestion are not handled in a timely manner, they will seriously threaten the personal safety of the public. In traffic situations, the speed and precision of vehicle detection are pivotal factors influencing traffic management. With the popularity of surveillance cameras and the significant development of Intelligent Traffic System (ITS), the expedient utilization of computer technology for the rapid processing of video and image data from road surveillance has emerged as a paramount imperative for actualizing ITS objectives [2,3].

Machine vision technology has a powerful video data processing capability, from which it can extract key information, such as vehicle color, model, brand, and license plate number [4,5,6]. This information enables the transportation department to grasp the road conditions in real time, e.g., the supervisory department can use this information to accurately identify various sorts of motor vehicles on the road, thus enhancing the monitoring of dangerous vehicles. In addition, machine vision technology can help accurately identify and locate these specific vehicles, providing strong support for the prevention of traffic accidents or criminal behavior.

In pursuit of more insightful insights, a multitude of researchers have employed various methodologies for vehicle detection and classification. Traditional vehicle detection methods traverse and scan the image through a fixed window and determine whether the current window contains the target vehicle based on manually designed features and classifiers, such as those proposed by Wang et al. [7], who utilized a pseudo-visual search mechanism to eliminate environmental interference in the image. Additionally, they integrated directional gradient histograms with local binary pattern fusion to enhance vehicle feature extraction capabilities. However, the process of manually annotating features is complicated and lacks real-time performance. As deep learning continues to advance, numerous researchers have employed deep learning techniques within the domain of vehicle detection. Currently, target detection based on deep learning can be categorized into the following two types: two-stage detection algorithms and single-stage detection algorithms. Most of the two-stage detection algorithms rely on the region candidate network to generate candidate frames, and feature extraction of the candidate frame target is carried out by a convolutional neural network [8]. For example, for a traditional deep learning network in which the feature information transfer process lacks interdependence, Ke et al. [9] proposed a dense attention network structure through the introduction of dense connections as well as an attention module that enhances the detection ability of the model. Gu et al. [10] introduced an enhanced Faster RCNN vehicle detection algorithm that aims to enhance the detection accuracy of various vehicle types by developing distinct scales of receptive fields for concurrent detection of image targets. Such algorithms demonstrate elevated detection accuracy but show limitations in real-time performance. The single-stage detection algorithms, on the other hand, discard region selection and directly recognize the target to be detected in the image; representative algorithms include the Single-Shot MultiBox Detector (SSD) [11], the You Only Look Once (YOLO) series [12,13,14], and EfficientDet [15]. In contrast to two-stage detection algorithms, single-stage detection algorithms exhibit superior real-time performance, but the detection accuracy is slightly lower [16].

Many scholars have conducted extensive and in-depth research in the field of lightweight networks and vehicle detection [17,18,19]. Chen et al. [20] proposed an efficient detection network that achieves three times the detection speed of YOLOv3 by fusing the advantages of densely connected networks and separable convolutions. Dong et al. [21] devised an advanced approach for vehicle detection, leveraging the C3Ghost module within the YOLOv5 neck network to streamline model parameters. Additionally, they bolstered detection accuracy through the integration of CBAM attention mechanisms and optimized the loss function to expedite model training. Zhang et al. [22] enhanced the YOLOv8 model by augmenting its feature fusion capabilities through multi-scale fusion within the backbone network. Additionally, they introduced a TA attention mechanism in the feature extraction phase to bolster model accuracy. Luo et al. [23] introduced an enhanced real-time detection model based on YOLOv5s to address the challenges posed by the high complexity and computational demands of vehicle detection models. This was achieved by incorporating a large-scale convolution function to amalgamate information from various feature images and optimizing the original spatial pyramid structure to bolster the model’s information extraction capabilities. However, as model accuracy increased, detection time also saw a gradual rise.

The above research has promoted the development of vehicle detection. However, the demanding real-time constraints within traffic scenarios pose a challenge, as existing algorithms struggle to strike a balance between detection speed and precision. To address this issue, the present paper introduces an enhanced real-time detection algorithm utilizing You Only Look Once version 7 (YOLOv7). This algorithm effectively reduces model parameters while ensuring recognition accuracy, thereby enabling deployment on edge devices.

The primary advancements delineated in this paper include the following:

⮚ Lightweight Modules: This paper employs the lightweight MobileNetV3 architecture to replace the backbone network of YOLOv7 and modifies the spatial pyramid parallel pooling structure to serial pooling to speed up the detection rate. Furthermore, inspired by the Generalized Sparse Convolution (GSConv) module, it utilizes GSConv to replace the standard convolution in the neck network. This neck network, in combination with the Spatial Pyramid Pooling Fast Cross-Stage Partial Channel (SPPFCSPC) module, forms the SPPCSPC-GS module, aiming to reduce the number of parameters in the model.
⮚ Attention Mechanisms Module: Aiming at the problem of decreasing feature extraction ability after the model is lightweighted, this paper enhances the detection accuracy of the model without substantially escalating the number of parameters by incorporating the coordinate attention (CA) mechanism in different feature layers.
⮚ Minimum Point Distance Intersection over Union (MPDIoU) Loss Function: In order to refine the detection speed of the model and reduce the bounding-box regression loss, the initial complete intersection over union (CIoU) loss function is substituted with the MPDIoU loss function.

## 2. Materials and Methods

In recent years, all kinds of image detection algorithms have performed extremely well on the metric of accuracy but have neglected the problem of model parameterization. Aside from accuracy, real-time performance is also a significant metric for evaluating models. Overly complex network models are hard to deploy on mobile devices with restricted computational resources and are also difficult to apply to scenarios with high real-time requirements. This paper aims to devise a lightweight and readily deployable network model, prioritizing the reduction in model parameters while preserving the algorithm’s accuracy.

### 2.1. YOLOV7 Network Structure

After multiple iterations of updates, the YOLO network model has given rise to the YOLOv7 model, which primarily comprises the following three modules: input, backbone, and head modules. The input module resizes the input image to a specified dimension to align with the input criteria of the backbone network. The backbone incorporates a CBS module, E-ELAN module, and MPConv module. E-ELAN is an efficient layer aggregation network that continuously improves the model’s learning ability without changing the structure of the transition layer. CBS convolutional layers improve the training efficiency and performance of the model by introducing binary supervision signals, while MPConv convolutional layers incorporate maxpool layers into their structure, forming upper and lower branches to effectively retain the most significant features. To adapt to multi-scale inputs, the head network uses a Spatial Pyramid Pooling (SPP) structure. To integrate features across various levels, an aggregated feature pyramid network structure is used to pass bottom-layer information to higher layers. Lastly, the reparameterized convolution (REPcon) structure modifies the channel counts of features at varying scales, enabling efficient feature representation.

### 2.2. YOLOV7 Improvements

**Figure 1:**
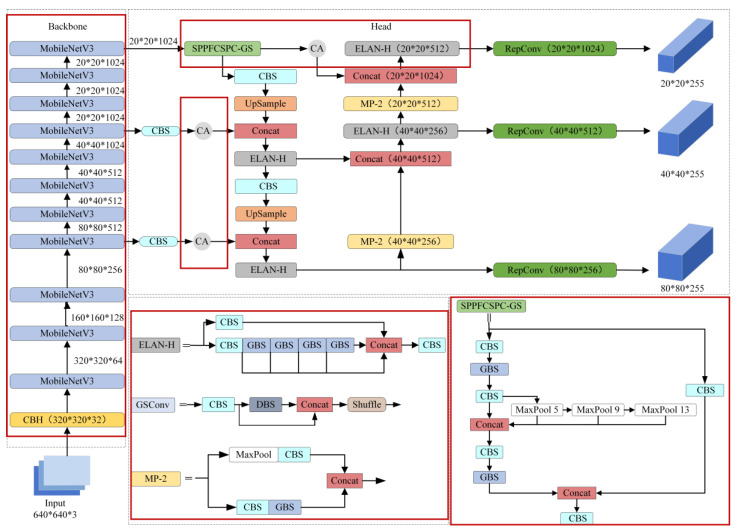
Improved YOLOv7 structural diagram.

The original model’s backbone network utilizes the DarkNet53 architecture, incorporating numerous residual structures that may escalate the model’s complexity and computational demands. Consequently, to address the issue of high parameter count and computational complexity in the original YOLOv7 network, which hinders deployment on terminal devices, this study undertakes a lightweight redesign of the network architecture [24]. The lightweight MobileNetV3 backbone network is employed instead of the DarkNet53 network to extract feature information from input images efficiently. Drawing inspiration from the Spatial Pyramid Pooling-Fast (SPPF) concept, the SPP module in the neck network is enhanced by adjusting the number of specified convolutional kernels. The original parallel pooling structure is transformed into a serial pooling structure, accelerating data processing and enhancing model training speed and feature extraction capability while keeping the sensory field intact. Furthermore, the conventional 3-convolutional kernel layer in SPPFCSPC is replaced with a lightweight GSConv layer, thereby forming the Spatial Pyramid Pooling Fast Cross-Stage Partial Channel-Generalized Sparse Convolution (SPPFCSPC-GS) module to further refine the model’s real-time performance. To counteract potential accuracy loss resulting from lightweight modifications, the CA mechanism is integrated into various feature extraction layers. The MPDIoU loss function is employed to refine the model’s representation of target features and enhance target detection accuracy. Figure 1 illustrates the enhanced architecture of the YOLOv7 network.

#### 2.2.1. Lightweight MobileNetV3 Module

**Figure 2:**
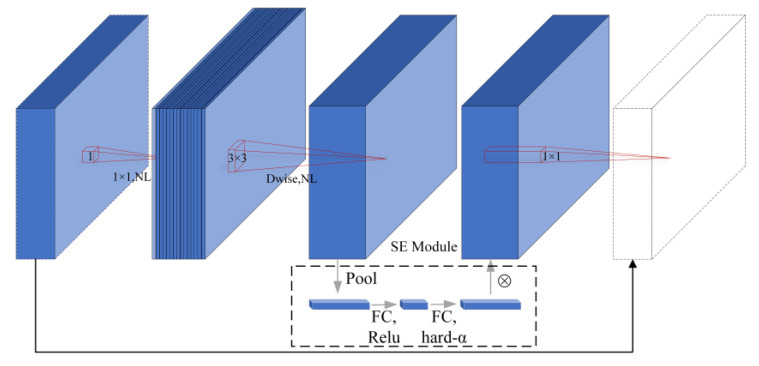
Structure of the bneck module for the MobileNetV3 model.

MobileNet is a series of Convolutional Neural Network (CNN) architectures for image classification proposed by a team of Google researchers [25]. Through the introduction of different versions, MobileNet introduces a series of innovative concepts with the main goal of decreasing the number of model parameters to increase operational efficiency on mobile devices while maintaining high classification accuracy. MobileNetV3 not only retains the inverted residual module and depth-separable convolution of MobileNetV1 and MobileNetV2 to optimize the network parameters but also introduces the Squeeze-and-Excite (SE) structure. It replaces the original swish activation function with the h-swish activation function to decrease the number of operations and optimizes the network structure to enhance model performance. The structure of the bneck module for the MobileNetV3 model is presented in Figure 2.

**Figure 3:**
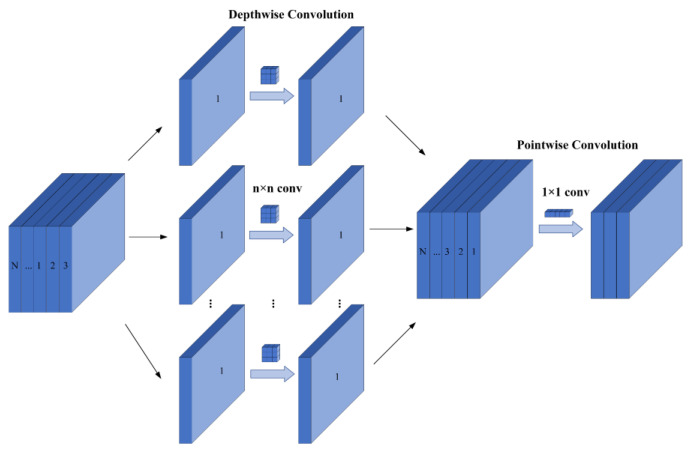
Depth-separable convolutional architecture diagram.

Figure 3 shows the structure of depth-separable convolution, which consists of depthwise (DW) convolution and pointwise (PW) convolution. DW convolution is a single-channel computation method, while PW convolution extracts the features of each element after DW convolution using a 1 × 1 convolution kernel. The relationship between its parameter calculation and ordinary convolution is shown below.

(1) $$\frac{D_{k}\times D_{k}\times 1\times M\times D_{F}\times D_{F}+1\times 1\times M\times N\times D_{F}\times D_{F}}{D_{k}\times D_{k}\times M\times N\times D_{F}\times D_{F}}=\frac{1}{N}+\frac{1}{D_{k}^{2}}$$

where M is the number of channels, $D_{k}\times D_{k}$ is the convolution kernel size, $N\times D_{F}\times D_{F}$ is the dimension of the output feature map, $D_{k}\times D_{k}\times 1\times M\times D_{F}\times D_{F}$ is the computation of a single ordinary convolution, and $1\times 1\times M\times N\times D_{F}\times D_{F}$ is the computation of PW.

According to Equation (1), when the convolution kernel is 3 × 3, the number of parameters in ordinary convolution is nine times greater than that of depth-separable convolution. This substitution not only decreases storage space and computational requirements but also lowers the hardware demands of the algorithm.

#### 2.2.2. SPPFCSPC-GS Module

**Figure 4:**
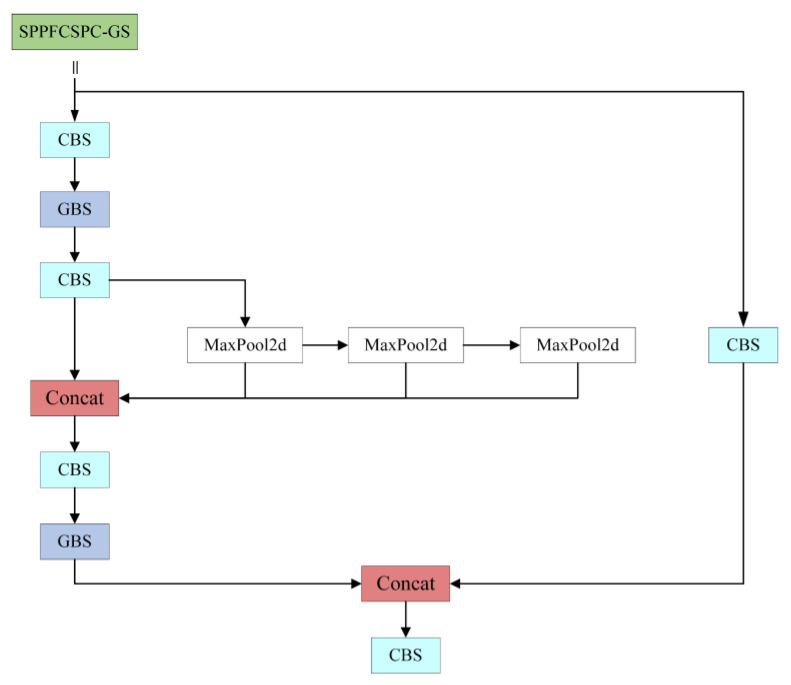
SPPFCSPC-GS module structure diagram.

The SPPFCSPC module draws inspiration from the concept of SPPF [26,27], which structurally reduces the number of times the convolution kernel size needs to be specified. While SPP requires specification of the dimensions of the convolution kernel three times to pool and splice the data from the CBS module, SPPF only requires specification of one convolution kernel. Additionally, each pooling operation’s output is utilized as the input for the subsequent pooling, accelerating data processing. This allows the model to enhance feature extraction from the data while keeping the receptive domain unchanged. Simultaneously, due to the depthwise-separable convolution’s channel-by-channel convolution, it loses plenty of information, which leads to low feature extraction ability. Therefore, this paper introduces a lightweight convolution layer, GSConv, which can effectively decrease the model parameters without affecting detection precision by fusing depthwise separable convolution with ordinary convolution, further improving the generalization ability of the model. Figure 4 illustrates the structure of the SPPFCSPC-GS.

**Figure 5:**
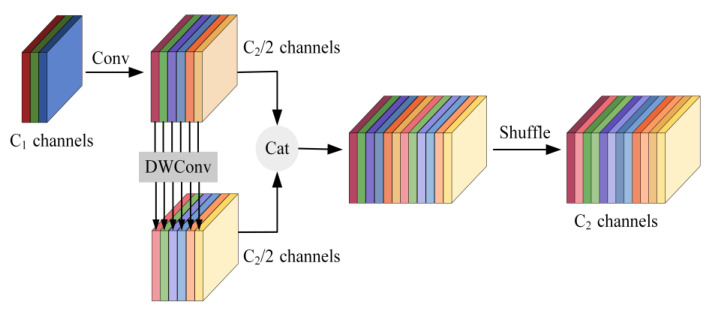
GSConv structure diagram.

In the figure, the GSConv module [28] splits the number of C1 channels in half by performing a 1 × 1 convolution on the input image and subsequently performs a 5 × 1 depth-separable convolution on the feature image so that the number of output channels remains half of the total number intended for the final output. Ultimately, it obtains a feature image with C2 output channels by integrating and rearranging the feature image. Figure 5 illustrates the architecture.

If the lightweight GSConv replaces all the ordinary convolutions in the model, it will increase the number of network layers of the network, exacerbate the resistance to data flow, and affect the inference speed of the model. While the neck network channel dimension is maximized and no transformation is needed, in this paper, the ordinary 3 convolutional kernels in the neck network are replaced with the lightweight GSConv convolutional layer, which lowers the computational burden and further enhances the generalization capacity of the model.

#### 2.2.3. CA Module

The integration of lightweight modules like MobileNetV3 in the model reduces the computational load and parameter count, albeit at the cost of decreased feature extraction capability. To amplify the network’s feature extraction prowess, an attention mechanism module is incorporated into the convolutional network. Among the commonly used lightweight attention mechanisms are SE and the Convolutional Block Attention Module (CBAM). While the SE mechanism focuses solely on channel attention, it neglects spatial dimensions, whereas the CBAM mechanism contemplates attention in both spatial and channel dimensions [29]. However, CBAM’s practical application complexity and computational resource consumption are relatively high [30]. Hence, when selecting an appropriate attention mechanism, it is essential to strike a balance based on specific task requirements and resource constraints.

**Figure 6:**
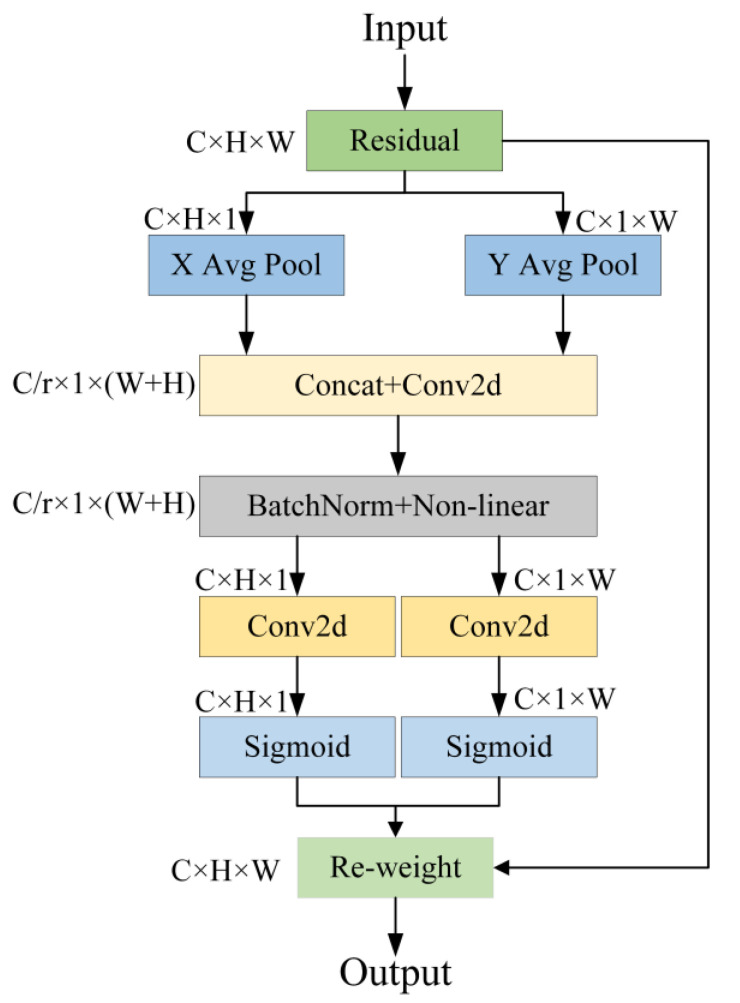
CA structure chart.

In order to amplify the original model structure’s ability to perceive the target locations within the feature map, this study employs the CA mechanism to strengthen the detector’s feature extraction aptitude for vehicles [31]. This is achieved by embedding position information into the channel attention as a means of attaining lightweight global attention. Figure 6 illustrates the architecture.

As shown in the figure, the CA mechanism first performs global pooling of the input feature map along the two directions of height and width. This leads to a feature map measuring H in height and W in width, where each channel is encoded. Subsequently, the feature maps from the two distinct directions are concatenated together. Then, a 1 × 1 convolution is applied to downscale the feature maps to C/r, and the downscaled feature maps are batch-normalized. After applying the Sigmoid activation function, we obtain a set of feature maps with dimensions of C/r × 1 × (W + H). Next, the feature map undergoes segmentation along the spatial dimension, and an additional 1 × 1 convolution is employed to derive weights in both directions. Ultimately, the attention weights are applied to the original image features via the activation function, yielding the final output feature map.

By generating weights in different directions for the feature map, the CA mechanism can focus on more important feature information. It not only grasps positional details across channels but also extracts position-specific information, assigning higher weights to significant pixel coordinates. This approach is instrumental in enhancing the precision of detection.

#### 2.2.4. MPDIoU Loss Function

The loss function serves to quantify the disparity between predicted and actual values of a model, with a lower value indicating greater robustness of the model. The initial YOLOv7 architecture employs the CIoU loss function, an extension of the Distance Intersection over Union (DIoU) loss, to gauge the loss associated with box scaling [32,33]. This method considers the overlap area, distance from the center point, and aspect ratio of the three geometric parameters, aiming to refine box predictions to values closer to actual dimensions. However, it fails to address the challenge of balancing between complex and straightforward samples. This potentially results in increased computational overhead during training and slows down the model’s convergence speed. The *CIoU* calculation formula is as follows:

(2) $$CIoU=IoU-\left(\frac{\rho^{2}\left(b,b^{gt}\right)}{c^{2}}+\alpha \nu \right)$$

(3) $$\nu =\frac{4}{\pi^{2}}{\left(\arctan\frac{w^{gt}}{h^{gt}}-\arctan\frac{w}{h}\right)}^{2}$$

(4) $$\alpha =\frac{\nu }{\left(1-IoU\right)+\nu }$$

where α and ν denote the aspect ratio; w and h denote the width and height of the prediction frame, respectively; $w^{gt}$ and $h^{gt}$ denote the width and height of the real frame, respectively; and $\rho^{2}\left(b,b^{gt}\right)$ represents the Euclidean distance between the prediction frame and the center point of the actual frame.

**Figure 7:**
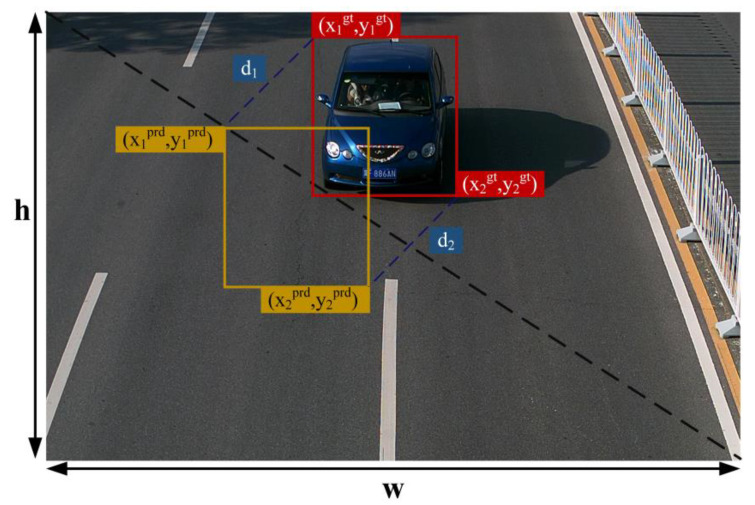
MPDIoU structure diagram.

To accelerate the training process and enhance the model’s classification accuracy, the CIoU is supplanted with the MPDIoU loss function [34]. MPDIoU is a comparative measure of the similarity of the bounding box with the minimum point distance. Figure 7 illustrates the architecture of MPDIoU. The red box indicates the actual bounding box, while the yellow box signifies the predicted bounding box. By minimizing the distance between the two corner points of the prediction, the loss is reduced. This method effectively resolves the issue where the existing loss function struggles to optimize effectively when the predicted bounding box and the ground-truth bounding box have identical aspect ratios but vastly different length and width values. It not only covers the advantages of the existing IoU and the paradigm loss but also makes up for the shortcomings of the existing loss, effectively reduces the localization loss, and further improves the prediction accuracy. *MPDIoU* is calculated as follows:

(5) $$d_{1}^{2}={\left(x_{1}^{prd}-x_{1}^{gt}\right)}^{2}+{\left(y_{1}^{prd}-y_{1}^{gt}\right)}^{2}$$

(6) $$d_{2}^{2}={\left(x_{2}^{prd}-x_{2}^{gt}\right)}^{2}+{\left(y_{2}^{prd}-y_{2}^{gt}\right)}^{2}$$

(7) $$MPDIoU=IoU-\frac{d_{1}^{2}}{w^{2}+h^{2}}-\frac{d_{2}^{2}}{w^{2}+h^{2}}$$

(8) $$L_{MPDIoU}=1-MPDIoU$$

where $d_{1}$ represents the distance between the predicted frame and the upper-left corner of the actual frame, and $d_{2}$ represents the distance between the predicted frame and the lower-right corner of the actual frame.

## 3. Experiment

### 3.1. Dataset

**Figure 8:**
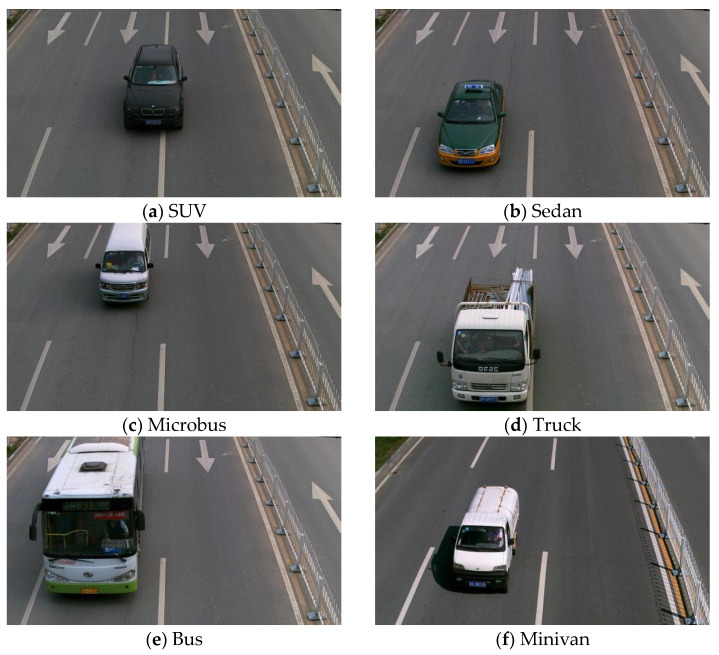
Images of various models.

In this paper, the BIT-Vehicle vehicle dataset released by the Beijing Institute of Technology is used [35], which has six model categories, namely SUV, Sedan, Microbus, Truck, Bus and Minivan. Table 1 provides the count of images in each labeling category, and the images of the models are presented in Figure 8. The dataset comprises 9850 images, of which 8865 are randomly selected for the training set at a ratio of 9:1, with the remaining 985 images allocated for the test set.

### 3.2. Experimental Environment

The experiments were conducted on a Win10 Professional operating system, using Python3.9.11 compiled language, Pytorch1.18.1 deep learning framework, and CUDA11.8 environment architecture. The hardware environment consisted of CPU Intel(R) w-2223 and two NVIDIA GeForce RTX2080Ti graphics cards (each with 11G video memory). During the training process, the model underwent iterative training utilizing the stochastic gradient descent (SGD) optimizer to optimize its parameters. A total of 200 rounds of training were performed, with the batch size set to 16, an initial learning rate of 0.01, the weight decay parameter set to 0.005, and a momentum parameter of 0.937.

### 3.3. Evaluation Metrics

To objectively and accurately assess the efficacy of the method proposed in this paper, the algorithm is evaluated using metrics including Precision (*P*), Recall (*R*), Parameters, mean Average Precision (*mAP*), and Frames Per Second (*FPS*), as illustrated by the following formula:

(9) $$P=\frac{TP}{TP+FP}$$

(10) $$AP=\int_{0}^{1}P(R)dR$$

(11) $$mAP=\frac{1}{n}\sum_{i=1}^{n}AP_{i}$$

(12) $$R=\frac{TP}{TP+FN}$$

(13) $$FPS=\frac{1}{T}$$

where TP represents the number of correctly predicted positive samples, FN signifies the number of incorrectly predicted negative samples, and FP indicates the number of incorrectly predicted positive samples; AP stands for the accuracy value of a single detected object, while mAP denotes the average accuracy across all categories, and n denotes the number of detected categories; T is the model preprocessing time.

## 4. Experimental Results and Analysis

### 4.1. Experimental Analysis of Adding the MPDIoU Loss Function

**Figure 9:**
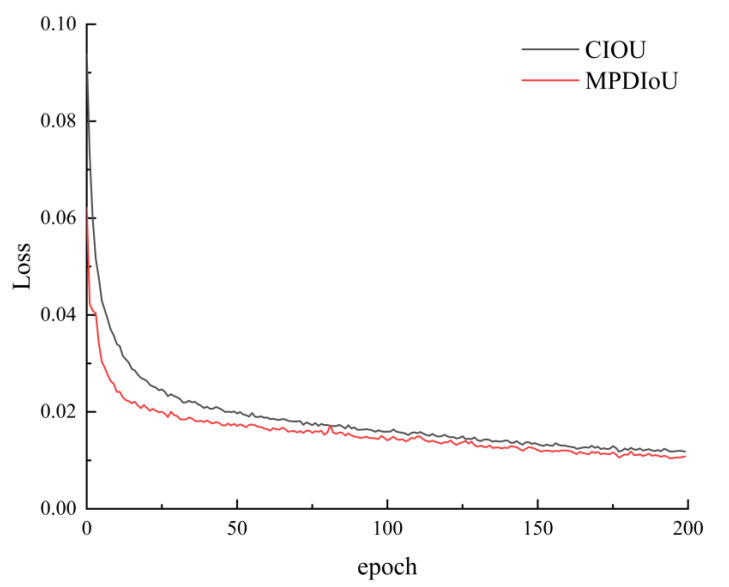
Loss curve comparison.

In this paper, the training loss of the enhanced model is contrasted with that of the original model; the loss curve is shown in Figure 9.

The x-axis represents the training epoch, while the y-axis represents the value of the loss function. It is apparent from the figure that the loss value experiences a rapid decline during the initial period. As the epoch increases, the loss value tends to stabilize. Furthermore, compared to the loss value of the original network, the loss value of the improved network is decreased. A lower loss value indicates a more effective model, suggesting a smaller prediction error across the entire dataset.

Table 2 provides a more intuitive understanding of the influence of the two loss functions on the model’s performance. The incorporation of the MPDIOU loss function leads to an enhancement in the model’s recall, signifying an augmentation in the detection of positive samples by the model. This illustrates that the enhanced model improves the detection accuracy of the original model.

### 4.2. Experimental Analysis of Improved Feature Pyramid Networks

To demonstrate more intuitively the impact of the SPPFCSPC-GS module in enhancing the detection speed of the model, comparison experiments are conducted using different SPP modules. The experimental findings are detailed in Table 3.

As evident from the table, integrating the SPPFCSPC module boosts the model’s training velocity and decreases the parameter count by 15.5%. However, there is a slight decrease in the mAP value. Subsequently, the introduction of GSConv convolution in the SPPFCSPC module not only further decreases the number of parameters of the model by 25.1% but also compensates for the slight decrease in the mAP value. Additionally, the convergence speed is improved compared to the original structure.

### 4.3. Overall Analysis

#### 4.3.1. Ablation Experiments

To verify the efficacy of the enhancement strategy outlined in this paper on the network model, the YOLOv7 algorithm is employed as the baseline model. Ablation experiments are conducted using the improvement strategy on the BIT-Vehicle dataset, and the outcomes of these trials are showcased in Table 4, where “√” indicates integration into the module (not integrated into the module otherwise).

According to Table 4, using the MobileNetV3 network to replace the original backbone network, the accuracy decreases by 0.4% compared to the YOLOv7 model, but the amount of model parameters decreases by 32.3%, and the FPS increases up to 93.5. It shows that the MobileNetV3 network can better balance real-time performance and accuracy, making it easy to subsequently deploy in edge devices. Meanwhile, the neck pyramid structure is improved, and the lightweight convolutional GSConv is incorporated to further increase the detection rate. This leads to a 46.0% increase in FPS and a 53.7% reduction in the number of model parameters in comparison to the primary model. With a decrease in the number of parameters, the model’s capability to extract features from the data also diminishes. However, this ability is improved by incorporating the CA mechanism into different feature extraction layers. From the group C experiments, it is evident that the model’s accuracy is enhanced to some extent, albeit with a marginal increase in the number of parameters. Finally, the MPDIoU loss function is incorporated to further augment the model’s detection accuracy and enhance its generalization capabilities. Compared with the original model, there is a minor improvement in accuracy, along with a reduction in the number of parameters by 52.8% and a 35.2% enhancement in FPS. This not only satisfies the stringent accuracy demands of the model but also aligns with the necessity to deploy it on edge devices.

#### 4.3.2. Comparison Experiments

To assess the efficacy of the proposed model, this paper conducts comparison experiments with prevalent target detection models such as CenterNet, YOLOv3, YOLOv5m, YOLOv7 and YOLOv8s. Additionally, to perform a more comprehensive analysis of the real-time detection performance of the enhanced models, further comparison trials are carried out using different lightweight backbone networks, specifically EfficientFormerv2 and EfficientVitM0. To maintain consistency in this experiment, all experiments are performed under identical equipment and environmental conditions, and the specific experimental results are showcased in Table 5.

As evident from Table 5, the single-stage unanchored frame CenterNet network has the highest parameter quantity. The YOLO series network model has a significantly reduced parameter quantity, but it still cannot meet the need for real-time detection. The YOLOv7 model demonstrates higher accuracy and has a lower model parameter quantity, so this paper considers YOLOv7 as the initial model for improvement. Compared with YOLOv3 and YOLOv5m, the amount of parameters is reduced by 71.4% and 29.9%, respectively, while the FPS is enhanced by 36.2% and 19.1%, respectively. In contrast to the most recent model, YOLOv8s, despite having a slightly larger number of parameters, the model introduced in this paper is more accurate and can be better applied to real scenarios. Compared to the lightweight efficient backbone network, the enhanced model presented in this paper demonstrates superior performance. In summary, the model presented in this paper showcases notable strengths in vehicle detection accuracy. The improved model not only achieves lightweight characteristics but also enhances detection accuracy, striking a better balance between detection precision and speed. This serves to further confirm the efficacy and trustworthiness of the algorithm proposed within this paper.

## 5. Conclusions

Due to the extensive number of parameters within the target detection model and the complexity of computations within the traffic scene, this paper proposes a lightweight design inspired by the original YOLOv7 model. The backbone network’s lightweighting is enhanced by incorporating the MobileNetV3 module, followed by substituting conventional convolutions in the neck structure with GSConv, thereby further reducing the model’s parameter count. To mitigate any potential decrease in model accuracy resulting from lightweighting, the model’s feature extraction capability is optimized by integrating the CA mechanism into the feature layer. This enhancement serves to improve the model’s overall detection performance. The loss function CIoU is substituted with MPDIoU to further refine the model’s training process and enhance classification accuracy. The experimental results indicate that, in contrast to the primitive YOLOv7 and other detection models, the improved model reduces the number of parameters in the model and improves the detection speed without compromising detection accuracy. The model achieves an exceptional equilibrium between detection accuracy and speed, a trait that renders it highly compatible for deployment in resource-limited embedded devices scenarios and provides strong support for performance optimization in practical applications, thus laying a foundation for the realization of intelligent traffic management.

Although the vehicle detection model designed in this paper better balances accuracy and real-time issues, there are still some aspects to be improved. For instance, the current dataset contains fewer vehicle categories. If an unlabeled vehicle category is detected, the model may misjudge. Subsequent research will focus on expanding the data categories to further improve experimental scenarios.

If the model is employed for the direction of autonomous driving, it can assist in improving the reaction speed and accuracy of autonomous driving vehicles. However, in actual application scenarios, there are still numerous limitations. Complex lighting conditions, severe vehicle occlusion, and different monitoring perspectives will affect the detection performance of the model. Therefore, it is crucial to consider a range of complex or extreme environmental factors prior to actual deployment to ensure that the model can better handle unexpected situations and complex traffic environments.

## Figures and Tables

**Table 1:** Number of labels by category.

**Category**	SUV	Sedan	Microbus	Truck	Bus	Minivan
**Number**	1392	5922	883	822	558	476

**Table 2:** Experimental results with different loss functions.

Model	mAP/%	FPS	P	R	AP/%					
					**SUV**	Sedan	Microbus	Truck	Bus	Minivan
**CIoU**	97.9	78.7	95.3	96.2	97.3	99.4	97.2	95.3	98.7	99.4
**MPDIoU**	98.2	78.9	95.3	96.5	96.9	99.4	98.5	97.2	99.4	97.9

**Table 3:** Experimental findings for different pyramid pooling structures.

Model	FPS	Parameters	mAP/%
SPPCSPC	78.7	37,304,436	97.9
SPPFCSPC	83.7	31,521,894	97.6
SPPFCSPC-GS	83.5	27,954,886	97.8

**Table 4:** Results of the ablation experiments.

Model	MobileNetV3	SPPFCSPC-GS	CA	MPDIoU	FPS	Parameters	mAP/%
YOLOv7					78.7	37,304,436	97.9
A	√				93.5	25,245,010	97.5
B	√	√			114.9	17,273,796	97.3
C	√	√	√		106.4	17,623,636	97.6
D	√	√	√	√	106.4	17,623,636	98.2

**Table 5:** Comparative experimental findings of different models.

Model	mAP/%	FPS	Parameters/M	AP/%					
				SUV	Sedan	Microbus	Truck	Bus	Minivan
CenterNet [36]	95.3	29.5	125.2	96.6	98.8	88.6	99.6	92.1	96.5
YOLOv3 [37]	97.3	78.1	61.6	97.7	99.4	95.3	94.0	98.5	98.8
YOLOv5m [38]	94.3	89.3	25.1	88.6	98.2	97.2	94.0	99.5	88.5
YOLOv7 [14]	97.9	78.7	37.3	97.3	99.4	97.3	95.4	98.7	99.4
EfficientFormerv2-YOLOv7 [39]	97.8	89.6	25.9	97.9	99.3	96.9	94.4	99.0	99.2
EfficientVitM0-YOLOv7 [40]	97.7	67.3	24.8	97.8	99.4	96.8	94.5	98.7	99.2
YOLOv8s [41]	94.8	123.3	13.2	88.9	97.7	96.4	93.8	99.5	92.6
Ours	98.2	106.4	17.6	97.4	99.3	98.3	97.0	99.1	97.8

## Data Availability

Available online: https://aistudio.baidu.com/datasetdetail/248053, (accessed on 28 March 2024).

## Abbreviations

ITS	Intelligent Traffic System
SSD	Single-Shot MultiBox Detector
YOLO	You Only Look Once
YOLOv7	You Only Look Once version 7
GSConv	Generalized-Sparse Convolution
CA	Coordinate Attention
MPDIoU	Minimum Point Distance Intersection over Union
CIoU	Complete Intersection over Union
SPP	Spatial Pyramid Pooling
REPcon	Reparameterized convolution
SPPF	Spatial Pyramid Pooling-Fast
SPPFCSPC	Spatial Pyramid Pooling Fast Cross-Stage Partial Channel
SPPFCSPC-GS	Spatial Pyramid Pooling Fast Cross-Stage Partial Channel-Generalized Sparse Convolution
CNN	Convolutional Neural Network
SE	Squeeze and Excite
DW	Depthwise
PW	Pointwise
CBAM	Convolutional Block Attention Module
DIoU	Distance Intersection over Union
P	Precision
R	Recall
mAP	mean Average Precision
FPS	Frames Per Second
SGD	Stochastic Gradient Descent
